# DNA Evidence Uncompromised by Active Oxygen

**DOI:** 10.1100/tsw.2010.47

**Published:** 2010-03-05

**Authors:** Ana Castelló, Francesc Francés, Fernando Verdú

**Affiliations:** Department of Legal and Forensic Medicine, Faculty of Medicine, University of Valencia, Spain

**Keywords:** forensic sciences, bloodstains investigation, presumptive test, hemoglobin test, forensic genetics

## Abstract

Currently, forensic sciences can make use of the potential of instrumental analysis techniques to obtain information from the smallest, even invisible, samples. However, as laboratory techniques improve, so too should the procedures applied in the search for and initial testing of clues in order to be equally effective. This requires continuous revision so that those procedures may resolve the problems that samples present. As far as bloodstains are concerned, there are methods available that are recognized as being both highly sensitive and effective. Nevertheless, the marketing of new cleaning products, those that contain active oxygen, has raised doubts about the ability of those procedures to detect blood. It has been shown that stains washed with these detergents (and still visible) invalidated both the presumptive test (reduced phenolphthalein, luminol, and Bluestar®) and that applied for determining human hemoglobin. These findings have caused considerable concern both within the forensic and scientific community, and among the general public, so obliging us to seek solutions. In this work, the effect of these new cleaning products on DNA analyses is studied. The results, encouraging ones, show that these detergents, despite invalidating all other tests, do not hinder the extraction, or the subsequent analysis, of DNA.

## INTRODUCTION

Impressive advances in instrumental analysis methods allow the smallest and even invisible samples to be analyzed. This fact, favorable in any field of science, is even more so in the field of criminal investigation, where it is commonplace to work with very little and often contaminated evidence.

However, to make use of the potential that laboratory analysis provides, it is essential to have the sufficiently sensitive means available for searching for clues and evidence so as to obtain the initial basic information on the sample type being worked on. These means are called the presumptive and confirmatory tests, respectively.

With regard to bloodstains, effective search and presumptive procedures[1,2] have been available for many years. Those using reduced phenolphthalein, luminol, and Bluestar®[3] are currently the most widely employed. These have been thoroughly studied and the possibilities of obtaining false-negative results are known[4,5,6,7,8,9].

Recently, however, a new problem has arisen: cleaning products that contain active oxygen have appeared on the market. These are detergents that do not contain hypochlorites, but are made with sodium percarbonate[10,11]. Tests undertaken on bloodstains washed with these products have provided worrying results (Castelló et al. 2009). After washing, although the stain remains visible, all the presumptive tests as well as the human hemoglobin test have given a negative result, i.e., apparently the stain is not a blood stain.

The article announcing these results caused deep concern among the scientific community. Headlines such as “Why hair bleach is a murderer’s best friend”[12] or “New detergent washes away stains of murder: study”[13], express the concern felt among forensic scientists and underlines the need to find a solution to this problem.

This work attempted to extract and analyze DNA from stains washed with active oxygen that had given negative results in the presumptive and human hemoglobin tests. The aim was to determine whether these cleaning products are also capable of hindering genetic analysis. For this purpose, the following work plan was employed.

## MATERIALS AND METHODS

### Material

A piece of cotton cloth was used as backing for bloodstains.

### Reagents

For the presumptive test, Phenolphthalein Dischaps™ (Sirchie, http://sirchie.com/SearchResult.aspx), luminol (3-aminophthalhydrazide) (Merck), sodium perborate (Panreac), sodium carbonate (Panreac), Bluestar, and distilled water were used.

The hemoglobin test was made using the Hexagon OBTI® test[14].

Finally, for DNA analysis, the necessary reagents were the following: Tris hydroxymethyl aminomethane (Panreac), hydrochloric acid (Panreac), ethylenediaminetetraacetic acid (Panreac), DL-dithiothreitol solution (Fluka), sodium chloride (Panreac), sodium dodecyl sulfate (Panreac), proteinase K (Sigma-Aldrich), phenol–chloroform–isoamyl alcohol (Sigma), agarose MS-8 (Promadisa), and 50xTAE (National Diagnostics).

### Sample Preparation[15]

Bloodstains were prepared using newly extracted blood samples without preservatives and the stains were made on the backing with five drops of blood. Having made the stains, they were left to dry at room temperature without any protection whatsoever. Drying time was initially set at 1 day. Subsequently, at different times (1, 5, 10, 20, and 30 days), the following procedure was undertaken:

- Four stains were cut out of the backing using a sterile scalpel. One of these was kept in the laboratory for subsequent use as a control. Another was washed using a product containing active oxygen (Neutrex™) whose composition, as appears on the label, is as follows: nonionic and anionic tensoactive agents, polycarboxylates, zeolites (concentration less than 5%), and sodium percarbonate (in a proportion greater than 30%). The pH of the product dissolved in water is 10.

- When washing, the manufacturer’s instructions were followed: the fabric was left in hot water (at 40ºC) containing the cleaning product for 2 h. Then it was rinsed in running water and left to dry over 24 h.
- The two remaining stains were used for control assays. One of them was washed in hot water (at 40ºC) without using detergent in order to determine the possible influence of water temperature on the results of the tests. The other was washed with active oxygen and cold water, with the aim of determining whether differences occur with the results obtained when using the product with hot water.

To confirm the results, the entire process was repeated five times for each of the periods (1, 5, 10, 20, and 30 days) using different samples.

## Presumptive Test and Human Hemoglobin Test

The presumptive tests (reduced phenolphthalein, luminol, and Bluestar) and the human hemoglobin test were undertaken on the dry stains, following the procedures described in the bibliography[15]. Having checked that all the results were negative, the stains were then processed for DNA extraction.

## DNA Extraction

A square of approximately 3 × 3 mm was cut from the stains using a sterile scalpel and inserted into appropriately marked microtubes, and 300 µl of extraction buffer (10 m*M* Tris–HCl, pH 8, 10 m*M* EDTA, pH 8, 2% SDS, 100 m*M* NaCl), 12 µl of 39 m*M* DTT, and 7.5 µl of proteinase K (10 mg/ml)[16,17] were added.

The tubes were incubated at 56ºC for 24 h. After incubation, DNA was extracted employing the phenol–chloroform method. The process was as follows:

- First, 300 µl of phenol–chloroform was added to each sample, and after agitating, was centrifuged at 13,000 rpm for 3 min. Then the liquid phase was transferred to a Microcon 100 filter (www.millipore.com ref 42413), where 100 µl of distilled water (filter buffer) had previously been added, and centrifuged at 13,000 rpm for 20 min.
- After removing the liquid, leaving only the filter in the tube, 200 µl of sterile distilled water were added – that is the washing process – and once again the sample was centrifuged at 13,000 rpm for 20 min.
- To recover the DNA, it was resuspended with 100 µl of distilled water and, as the last phase before inserting the filter downwards in new tubes, it was centrifuged at 13,000 rpm for 5 min.

## DNA Quantification

Before proceeding to amplification, checks were undertaken to determine whether DNA had been extracted, and in what quantity and quality.

For this purpose, the extraction product was submitted to two quantification processes. At the first, a horizontal electrophoresis in agarose gel of low electroendosmosis was used, at 0.8%. For each sample, 1:1, 1:5, and 1:25 dilutions were prepared. In addition, a control of 10 ng/pl concentration was added.

The results of the electrophoresis were observed under an ultraviolet lamp after having stained them with ethidium bromide.

A second quantification was made using pectrophotometry. For this, a NanoPhotometer™ (IMPLEN GmBH) was selected.

## PCR Amplification

Multiplex PCR with the AmpflSTR Profiler PCR amplification kit (Applied Biosystems) was performed at a final volume of 10 µl, composed of 6.6 µl of master mix (mixed 4.2 µl of PCR mix, 2.2 µl of primer set, and 0.2 µl of AmpliTaq Gold polymerase per tube) and 0.7 ng of sample DNA on a GeneAmp PCR System 9700 thermal cycler (Applied Biosystems), according to the manual.

## Electrophoresis

Amplified PCR products were run on an ABI PRISM 310 Genetic Analyzer and analyzed using GeneScan analysis software version 3.1 and Genotyper software version 2.5. Alleles were designated by comparison with the allelic ladder marker contained in the kit, according to the Amp-FlSTR User's Manual.

In order to confirm the results, the entire process was repeated three times for each of the periods (1, 5, 10, 20, and 30 days) using different samples.

## RESULTS

## Presumptive Test and Human Hemoglobin Test

The presumptive test and human hemoglobin test were negative for the stains analyzed.

## DNA Analysis

We were able to extract DNA from all the samples analyzed, and of a sufficient quantity and quality to obtain a genetic profile, coinciding with that of the control samples.

Fig. 1 shows the presence of the DNA extracted from the problem samples. From the image, a certain degradation can be observed that is due to the stains being treated with active oxygen, which is not observed in the controls. However, this effect has not hindered their subsequent amplification.

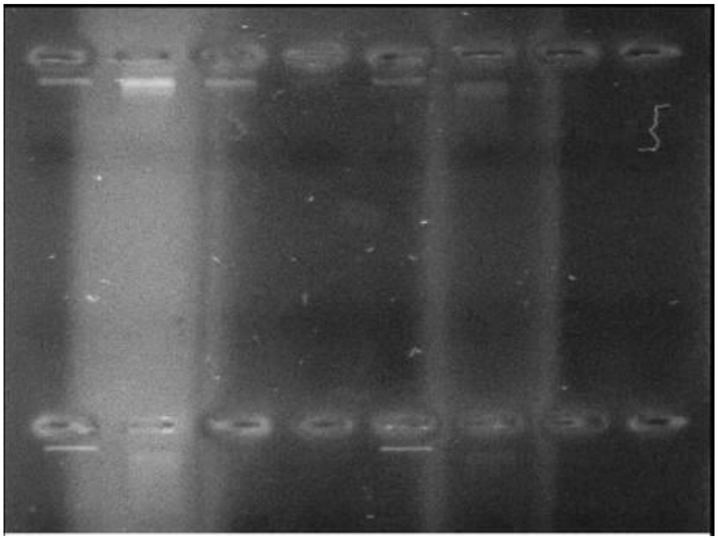

**FIGURE 1.**

The results on the quantity of DNA extracted from NanoPhotometer were coincident with those from the electrophoresis in gel. Nevertheless, it is not possible, through spectrophotometry, to observe the degradation of the DNA that is observable in the in-gel development. Table 1 shows the average quantities of DNA obtained from problem and control samples in each period (1, 5, 10, 20, and 30 days).

**TABLE 1**
**Average Quantities (Concentration) of DNA Obtained from Problem and Control Samples in Each Period**

| Time (Days) | DNA Concentrations (ng/µl) | | | |
|---|---|---|---|---|
| | Control Sample | Control Assay 1 | Control Assay 2 | Problem |
| 1 | 41 | 35 | 10 | 7 |
| 5 | 37 | 33 | 12 | 5 |
| 10 | 40 | 34 | 11 | 6 |
| 20 | 36 | 30 | 11 | 7 |
| 30 | 35 | 36 | 10 | 7 |

**Control sample:** Bloodstains not washed.

**Control assay 1:** Bloodstains washed in hot water (at 40ºC) without using detergent.

**Control assay 2:** Bloodstains washed with active oxygen and cold water.

**Problem:** Bloodstains washed with active oxygen and hot water.

## DISCUSSION

The results reaffirm the deleterious effect of new detergents on the blood search, presumptive, and confirmation procedures. However, although these initial tests are invalidated, DNA analysis is not impeded, as it remains positive in all the cases studied.

Consequently, faced with a suspicious stain, one has to think of the possibility that it has been washed with an active oxygen product. Then, even though everything indicates that it is not blood, it would be recommendable to try a DNA analysis.

Obviously invisible or latent stains cannot be identified when these products have been used. The evidence will be lost and this poses a problem that is yet to be solved. Perhaps in the future it will be.

In conclusion: active oxygen cannot doctor the evidence, at least, not completely.

## ACKNOWLEDGMENTS

We thank the staff of the Laboratory of Forensic Identification (University of Granada, Spain) and to the Genetic and Molecular Epidemiology Unit (University of Valencia, Spain) for their invaluable collaboration.
